# Hyperoxaluria, Hypocitraturia, Hypomagnesiuria, and Lack of Intestinal Colonization by *Oxalobacter formigenes* in a Cervical Spinal Cord Injury Patient with Suprapubic Cystostomy, Short Bowel, and Nephrolithiasis

**DOI:** 10.1100/tsw.2006.373

**Published:** 2006-04-06

**Authors:** Subramanian Vaidyanathan, Gerd E. von Unruh, Ian D. Watson, Norbert Laube, Steve Willets, Bakul L. Soni

**Affiliations:** ^1^ Regional Spinal Injuries Centre, Southport and Ormskirk Hospital NHS Trust, Southport, Merseyside PR8 6PN, UK; ^2^ Medizinische Universitätsklinik I, Sigmund-Freud-Str. 25, D 53105 Bonn, Germany; ^3^ Department of Clinical Biochemistry, Southport and Ormskirk Hospital NHS Trust, Town Lane, Southport, Merseyside PR8 6PN, UK; ^4^ Urologische Universitätsklinik, Experimentelle Urologie, Sigmund-Freud-Str.25, D 53105 Bonn, Germany

**Keywords:** spinal cord injury, kidney – calculi, Oxalobacter formigenes, hyperoxaluria, hypocitraturia

## Abstract

Although urolithiasis is common in spinal cord injury patients, it is presumed that the predisposing factors for urinary stones in spinal cord injury patients are immobilization-induced hypercalciuria in the initial period after spinal injury and, in later stages, urine infection by urease-producing micro-organisms, e.g., *Proteus* sp., which cause struvite stones. We describe a patient who sustained C-7 complete tetraplegia in a road traffic accident in 1970, when he was 16 years old. Left ureterolithotomy was performed in 1971 followed by left nephrectomy in 1972. Probably due to adhesions, this patient developed volvulus of the intestine in 1974. As he had complete tetraplegia, he did not feel pain in the abdomen and there was a delay in the diagnosis of volvulus, which led to ischemia of a large segment of the small bowel. All but 1 ft of jejunum and 1 ft of ileum were resected leaving the large bowel intact. In 1998, suprapubic cystostomy was performed. In 2004, this patient developed calculus in the solitary right kidney. Complete stone clearance was achieved by extracorporeal shock wave lithotripsy. Stone analysis: calcium oxalate 60% and calcium phosphate 40%. Metabolic evaluation revealed hyperoxaluria, hypocitraturia, and hypomagnesiuria. Since this patient had hyperoxaluria, the stool was tested for *Oxalobacter formigenes*, a specific oxalate-degrading, anerobic bacterium inhabiting the gastrointestinal tracts of humans; absence of this bacterium appears to be a risk factor for development of hyperoxaluria and, subsequently, calcium oxalate kidney stone disease. DNA from the stool was extracted using the QIAamp DNA stool Mini Kit (Qiagen, Chatsworth, CA). The genomic DNA was amplified by polymerase chain reaction using specific primers for oxc gene (developed by Sidhu and associates). The stool sample tested negative for *O. formigenes*. The patient was prescribed potassium citrate mixture; he was advised to avoid oxalate-rich food, maintain recommended levels of calcium in his diet, and take live bio-yogurt. Two months later, 24-h urinary oxalate decreased from 0.618 to 0.411 mmol/day; 24-h urine citrate increased from 0.58 to 1.10 mmol/day. Six months later, an oxalate absorption test was performed. The patient swallowed a capsule, soluble in gastric juice, containing 50 mg (0.37 mmol) sodium [^13^C_2_]oxalate corresponding to 33.8 mg of [^13^C_2_]oxalic acid. The amount of labeled oxalate, excreted in urine, was measured by a gas chromatographic-mass spectrometric assay. Oxalate absorption, expressed as the percentage of the labeled dose recovered in the 24-h urine after dosing, was 8.3% (reference range: 2.3–17.5%). In addition to other conventional measures, oral administration of *O. formigenes* or lactic acid bacteria mixture to promote bacterial degradation of oxalate in the gut, and thus combat hyperoxaluria, may play a role in prevention of calcium oxalate kidney stones.

## INTRODUCTION

In a recently published update on metabolic evaluation of stone formers, Pearle and Park[1] state that medical and dietary history, stone analysis, serologic tests, and urinalysis constitute the initial screening regimen in patients who have been diagnosed with urolithiasis, and describe a simple metabolic evaluation for first-time or low-risk stone formers. Metabolic evaluation and treatment has clearly been shown in randomized trials to reduce stone recurrence rates. Although urolithiasis is common in spinal cord injury patients, it is presumed that the predisposing factors for urinary stones in spinal cord injury patients are immobilization-induced hypercalciuria in the initial period after spinal injury and, in later stages, urine infection by urease-producing micro-organisms, e.g., *Proteus* sp., causing struvite stones. A review of 893 patients (followed up by the Milwaukee Veterans Administration Medical Centre from 1970 to 1984) showed that serum creatinine, phosphorus, uric acid, and calcium levels were similar in spinal cord injury patients with and without renal stones[2], but there was a relationship between sepsis, positive urine culture, and kidney stone formation.

We describe a cervical spinal cord injury patient with a single kidney and suprapubic cystostomy who developed renal calculus. Metabolic evaluation revealed hyperoxaluria, hypocitraturia, and hypomagnesiuria. This patient underwent tests for oxalate absorption and *Oxalobacter formigenes* in the stool. *O. formigenes* was not detected in the feces. We advised this patient to take potassium citrate, avoid oxalate-rich food, maintain recommended levels of calcium in his diet, and take bio-yogurt. We discuss the possible therapeutic role of manipulating intestinal flora, which might promote bacterial degradation of oxalate in the gut and thus combat hyperoxaluria.

## CASE REPORT

A 16-year-old boy sustained C-7 complete tetraplegia in a road traffic accident in 1970. Left ureterolithotomy was performed in 1971 followed by left nephrectomy in 1972. Probably due to adhesions, this patient developed volvulus of the intestine in 1974. As he had complete tetraplegia, he did not feel pain in the abdomen and there was a delay in the diagnosis of volvulus, which led to ischemia of a large segment of the small bowel. The surgeon had to remove all but 1 ft of jejunum and 1 ft of ileum. The large bowel was intact. In 1998, suprapubic cystostomy was performed. In 2004, this patient developed calculus in the solitary right kidney (Fig. 1). Ureteric stenting was performed, which was followed by extracorporeal shock wave lithotripsy of right renal calculus. Complete clearance was achieved.

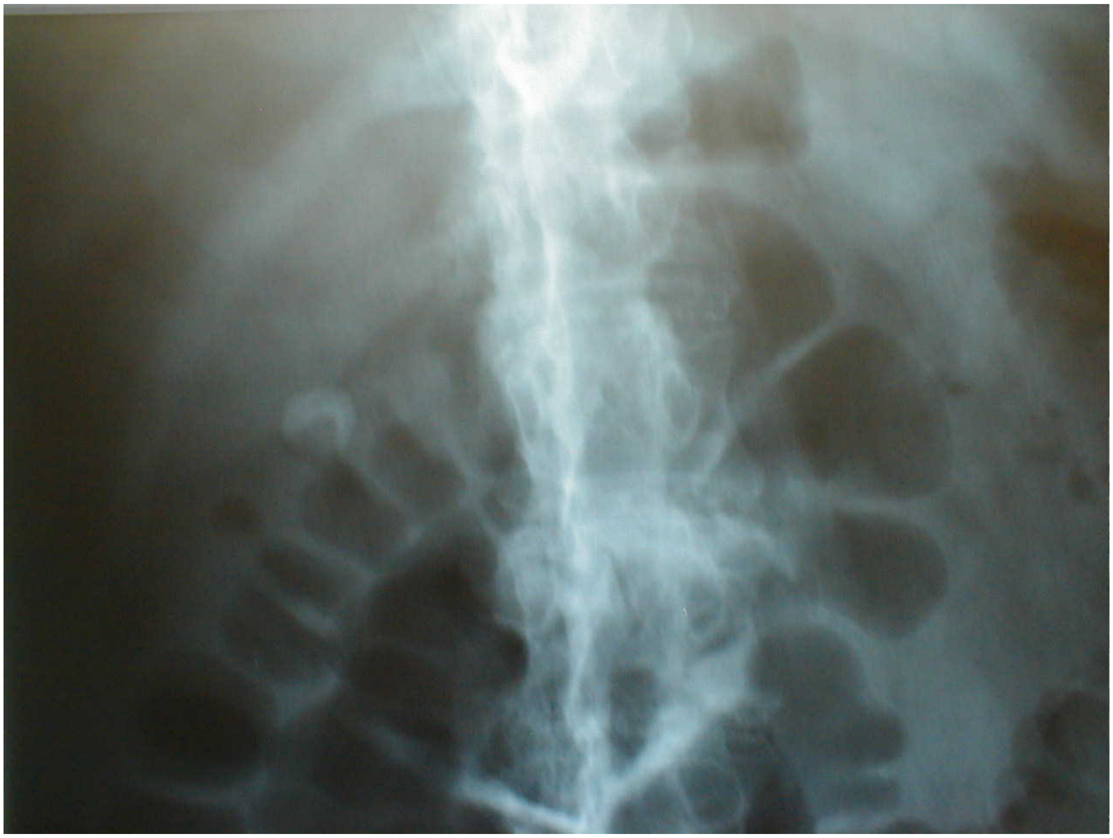

**FIGURE 1.** X-ray of abdomen, taken on 09 March 2004, showing a large radio-opaque calculus in right kidney.

## Laboratory Investigations

Urine microbiology showed growth of *Klebsiella* sp. and *Enterococcus faecalis*. Biochemical analysis of renal calculus revealed calcium oxalate 60% and calcium phosphate 40%. The 24-h urine oxalate was 0.618 mmol/day (reference range: 0.189–0.477 mmol/day), 24-h urine citrate was 0.58 mmol/day (reference range: 1.68–6.45 mmol/day), creatinine clearance was 51 ml/min, 24-h urine calcium was 2.03 mmol/day (reference range: 2.50–7.50 mmol/day), 24-h urine phosphate was 11.8 mmol/day (reference range: 12.9–42.0 mmol/day), and 24-h urine magnesium was 2 mmol/day (reference range: 3–5 mmol/day).

Serum chemistry results are as follow: Reference range is given in parentheses.

- Sodium — 140 mmol/l (133–146)
- Potassium — 3.9 mmol/l (3.6–5.2)
- Urea — 3.4 mmol/l (2.3–7.5)
- Creatinine — 63 umol/l (0–135)
- Chloride — 110 mmol/l (95–110)
- Bicarbonate — 25 mmol/l (22–30)
- Calcium — 2.46 mmol/l (2.20–2.60)

- Phosphate — 0.93 mmol/l (0.80–1.50)
- Magnesium — 1.08 mmol/l (0.70–1.00)
- Parathyroid hormone (PTH) — 4.5 pmol/l (1.1–6.9)

Summarizing, this patient exhibited hyperoxaluria, hypocitraturia, hypomagnesiuria, and normal blood levels of calcium, phosphate, and PTH.

## Test for *Oxalobacter formigenes* in Stool Sample

Since this patient had hyperoxaluria, his stool was tested for *O. formigenes*, a specific oxalate-degrading, anerobic bacterium inhabiting the gastrointestinal tracts of humans; absence of this bacterium appears to be a risk factor for development of hyperoxaluria and, subsequently, calcium oxalate kidney stone disease. A sample of the stool was tested for *O. formigenes* in the laboratory of Dr. Laube at the University of Bonn, Germany, by the method originally described by Sidhu and associates[3]. Polymerase chain reaction (PCR) was performed using LightCycler from Roche. DNA was extracted using the QIAamp DNA stool Mini Kit (Qiagen, Chatsworth, CA). The genomic DNA was amplified using specific primers for oxc gene, which were developed by Sidhu and associates. PCR of stool sample tested negative for *O. formigenes*.

## Medical Treatment Towards Prevention of Recurrent Kidney Stone

This patient had short bowel and hyperoxaluria; a sample of stool tested negative for *O. formigenes*. Therefore, he was advised to avoid oxalate-rich food as well as fatty food, and adhere to a nutritionally balanced diet, which included recommended levels of calcium and bio-yogurt containing *Lactobacillus acidophilus*, *Streptococcus thermophilus*, and bifidobacterium. He was also prescribed potassium citrate mixture (British Pharmacopoeia) 10 ml, three times daily, well diluted with water, by mouth. The composition of this oral solution was potassium citrate, 3 g; citric acid monohydrate, 500 mg; syrup, 2.5 ml; quillaia tincture, 0.1 ml; lemon spirit, 0.05 ml; double-strength chloroform water, 3 ml; water to 10 ml. This mixture contained about 28 mmol $K^+$/10 ml. Two months later, 24-h urinary oxalate decreased from 0.618 to 0.411 mmol/day; 24-h urine citrate increased from 0.58 to 1.10 mmol/day. Six months later, an oxalate absorption test was performed.

## Oxalate Absorption Test

Hyperoxaluria can result from increased endogenous production, from excessive oxalate content of the food, or from intestinal hyperabsorption. For a causal therapy, it is important to discriminate between metabolic and hyperabsorptive hyperoxaluria. Conventionally, in urine, the endogenous oxalate cannot be distinguished from food-derived oxalate. The $^{13}C$-oxalate test, as developed by von Unruh and associates[4], allows this differentiation. This oxalate absorption test applies administering physiological dose (50 mg disodium salt of [$^{13}C_2$]oxalic acid) of labeled oxalate by mouth. The patient is instructed to collect urine at intervals of 12 h on day 1 and in intervals of 6, 6, and 12 h on day 2. Strong (25%) hydrochloric acid is used to preserve the urine samples. The amount of 25% hydrochloric acid per day is 60 ml, distributed in two 30-ml portions (bottles I and II) for day 1; for day 2, 25% hydrochloric acid is used in two 15-ml portions for the 6-hour collection periods (bottles III and IV), and again a 30-ml portion for bottle V. The pH in the urine sample is always measured before work-up.

At 08:00 on day 2, a capsule, soluble in gastric juice, containing 50 mg (0.37 mmol) sodium [$^{13}C_2$]oxalate corresponding to 33.8 mg of [$^{13}C_2$]oxalic acid is ingested with water. Absorbed oxalate (labeled as well as unlabeled) is excreted rapidly and completely via the kidneys (biologic half-life: 1.5 h). The amount of labeled oxalate, excreted in urine, is measured by a gas chromatographic-mass spectrometric assay. Oxalate absorption is expressed as the percentage of the labeled dose recovered in the 24-h urine after dosing. Oxalate hyperabsorption is defined as an absorption exceeding 10%.

The oxalate absorption test showed that the patient had oxalate absorption of 8.3%, reference range being 2.3–17.5%, mean value 8%; 24-h urinary oxalate excretion was 0.385 and 0.390 mmol/24 h on day 1 and 2 of the test, respectively. By restricting oxalate-rich food as well as fatty food, and by taking potassium citrate mixture, 24-h urine oxalate excretion had decreased from 0.618 to 0.385 mmol/day.

## DISCUSSION

### Pathogenesis of Oxalate Nephrolithiasis in Patients with Short Bowel

This patient with short bowel developed a stone in the solitary kidney. Oxalate nephrolithiasis can occur following significant small bowel resection when any portion of the colon remains. Dietary oxalate normally passes harmlessly through the gut as an insoluble calcium salt. However, with steatorrhea, the calcium concentration drops because calcium preferentially binds to fatty acids and oxalate remains in a free form, which is absorbed by the colon, producing enteric hyperoxaluria.

From the extent of bowel resection this patient had, an oxalate absorption in the range of 30–40% would be expected in an able-bodied individual with normal colonic transit time. But less than the predicted high value for oxalate absorption was obtained in this patient, who had short bowel. This patient, who had tetraplegia and consequently neuropathic bowel, usually evacuated his bowels only thrice a week (Mondays, Wednesdays, and Fridays). Spinal cord injury results in severely prolonged colonic transit times both in the acute and chronic phase[5]. Impaired colonic motility, significantly prolonged segmental colorectal transit time of transverse colon and descending colon, infrequent bowel emptying, and consequent fecal loading of the colon might explain the anomaly in the result of the oxalate absorption test. It is possible that the oxalate, included in water-poor feces, will not be absorbed even if remaining in the colon for a long time.

### Treatment of Nephrolithiasis in Patients with Short Bowel

Patients with short bowel who develop hyperoxaluria are typically managed by restricting dietary fat and oxalate and by providing an oral calcium supplement, but one must be careful with calcium therapy and monitor it. Excessive calcium can actually increase the CaOx supersaturation in the urine and thus increase the risk of stone formation. Patients with refractory hyperoxaluria may benefit from cholestyramine, which limits bile salt injury to the colon. (Bile salts can make the colon more permeable to oxalate and thus facilitate oxalate absorption from the colon.)

Kato and associates[6] managed a 56-year-old woman with bilateral renal stones. This patient had undergone extensive resection of the small intestine due to strangulating obstructive ileus 7 years ago. She had residual intestine of only 20 cm. The stones were treated by extracorporeal shock wave lithotripsy. Urolithiasis was diagnosed to be due to enteric hyperoxaluria (103.8 mg/day) caused by short bowel syndrome. She was treated with oral calcium lactate, sodium/potassium citrate, and magnesium oxide. We advised our patient to adhere to a nutritionally balanced diet with the recommended intake of calcium and prescribed potassium citrate mixture by mouth.

### Rationale for Prescribing Oral Citrate and Magnesium to Patients with Hyperoxaluria, Hypocitraturia, and Hypomagnesiuria for Prevention of Oxalate Nephrolithiasis

Our patient had a renal calculus, which was composed predominantly of calcium oxalate. Although the stone was fragmented successfully by extracorporeal shock wave lithotripsy, we were concerned about possible recurrence of urolithiasis, as this patient had hyperoxaluria, hypomagnesiuria, and hypocitraturia. Hypocitraturia is an important risk factor for calcium oxalate nephrolithiasis[7]. Kato and associates[8] administered potassium-sodium citrate and magnesium oxide to 14 patients with recurrent calcium oxalate stones. After administration of both supplements to the patients with stones, the citrate, magnesium, and potassium levels in 24-h urine samples increased by 62.1, 63.3, and 25.3%, respectively, and oxalate decreased by 66.5%. These authors concluded that the combination of potassium-sodium citrate and magnesium oxide is more effective than either supplement alone in inhibiting the crystallization of calcium oxalate stones by improving the urinary parameters of patients with hypocitraturia and/or hypomagnesiuria. However, in a double-blind, randomized, placebo-controlled trial, there was no significant difference between recurrence rates with 650 or 1300 mg magnesium oxide daily and the placebo. Another trial reported 391 mg (21 mEq) magnesium daily as a mixed salt, magnesium potassium citrate, reduced calcium stone recurrence by 90%, similar to potassium citrate, but with better gastrointestinal tolerance[9]. Neither magnesium potassium citrate nor potassium-sodium citrate was available in the British National Formulary; therefore, we prescribed potassium citrate mixture to this patient.

## Importance of Nutritionally Balanced Diet in Prevention of Nephrolithiasis

Siener and associates[10] demonstrated that a nutritionally balanced diet significantly reduced stone-forming potential in men and women with calcium oxalate stone disease, although no change occurred in urinary oxalate and magnesium excretion. This patient was prescribed potassium citrate and was advised to adhere to a nutritionally balanced diet *avoiding* (1) oxalate-rich food, (2) low fluid intake, and (3) increased intake of protein and alcohol. Following nutritional intervention in our patient, 24-h urine oxalate excretion decreased from 0.618 to 0.385 mmol/day; 24-h urine citrate increased from 0.58 to 1.10 mmol/day.

## Biological Manipulation of Intestinal Flora in Treatment of Hyperoxaluria and Oxalate Nephrolithiasis

Oxalate is present in many foods and beverages. Bacterial, rather than host, enzymes are required for the intestinal degradation of oxalate in humans[11]. Intestinal oxalate-degrading bacteria are capable of degrading oxalate to $CO_2$ and formate, the latter being absorbed or further metabolized. *O. formigenes*, an obligate anerobic microbe normally found in the intestinal tract, contains two enzymes [formyl coA transferase (frc) and oxalyl-coenzyme A decarboxylase (oxc)] that allow it to utilize oxalate as an energy source, in the process converting oxalate to formate and $CO_2$, as well as a specific oxalate/formate antiporter (Ox1T). The oxalate-degrading enzyme oxalyl-CoA decarboxylase is also found in *Bifidobacterium lactis*. Lack of *O. formigenes* can result in higher absorption of oxalate, leading to an increased risk of calcium oxalate kidney stone formation[12].

Duncan and associates[13] administered *O. formigenes* by mouth to human volunteers and found a reduction in the amount of oxalate excreted during the 6 h immediately following ingestion of an oxalate load (from 3.0 ± 0.6 to 1.9 ± 0.1 mg/h). In the same tests, the ingestion of *O. formigenes* also decreased the urinary oxalate/creatinine ratio from 45.2 ± 9.9 to 27.0 ± 4. 2 mg/g. Hoppe and associates[14] found the oral application of *O. formigenes* successful in patients with primary hyperoxaluria. In one patient with constant intestinal colonization with *O. formigenes*, urinary oxalate excretion returned to normal level over time. Other bacteria with possible oxalate-degrading potency are lactic acid bacteria, as well as *Enterococcus faecalis* and *Eubacterium lentum*. Although no strain of *Lactobacillus acidophilus*, *L. plantarum*, *L. brevis*, *S. thermophilus*, or *B. infantis* expressed the oxalate/formate antiporter (Ox1T) gene, the urinary excretion of oxalate, which is a major risk factor for renal stone formation and growth in patients with calcium-oxalate urolithiasis, can be reduced with treatment using a high concentration of freeze-dried lactic acid bacteria[15]. Administration of lactic acid bacteria mixture (Oxadrop, VSL

Pharmaceuticals), one dose per day, to ten patients with chronic fat malabsorption, calcium oxalate stones, and hyperoxaluria, resulted in a decrease in mean urinary oxalate excretion by 19% after 1 month[16]. Such biological manipulation of the endogenous digestive microflora can be a novel approach for the prevention of urinary stone formation. Therapeutic administration of *O. formigenes* may ultimately provide the best practical approach for the prevention or alleviation of hyperoxaluria together with potassium citrate supplementation.

## Effect of Antibiotic Therapy on Intestinal Colonization with *Oxalobacter formigenes*

*O. formigenes* is assumed to be antibiotic sensitive and repeated antibiotic therapies could eradicate it. Mittal and associates[17] observed a direct association between antibiotic consumption and absence of *O. formigenes* in stool samples. Spinal cord injury patients are more susceptible to develop urine, chest, and soft tissue infections than able-bodied persons and, therefore, persons with spinal cord injury often receive prescriptions for antibiotics. However, frequent use of antibiotics may adversely affect intestinal colonization of *O. formigenes*. In children with cystic fibrosis, prolonged and widespread use of antibiotics induced a permanent decolonization of the intestine by *O. formigenes*. Only 1 of the 43 children with cystic fibrosis who were tested for *O. formigenes* had normal numbers of *O. formigenes* in stool samples; this patient with normal colonization by *O. formigenes* had *not* been treated with antibiotics[18]. Seven patients who were colonized with *O. formigenes* had normal urinary oxalate levels, but 19 (53%) of 36 patients who were not colonized with *O. formigenes* were hyperoxaluric. Absence of *O. formigenes* from the intestinal tract of children with cystic fibrosis appeared to lead to increased absorption of oxalate, thereby increasing the risk of hyperoxaluria. Such antibiotic-induced decolonization of the gut may occur in spinal cord injury patients as well. Troxel and associates[19] recommended the use of antibiotics such as penicillin or trimethoprim-sulfamethoxazole instead of quinolones (e.g., ciprofloxacin for urine infection) in calcium oxalate stone formers, since quinolones reduced the level of *O. formigenes* in the gut, whereas penicillin or trimethoprim-sulfamethoxazole, did not have any effect on the *O. formigenes* level in the gut.

## Therapeutic Care Pathway for Managing Hyperoxaluria in Spinal Cord Injury Patients

In patients who exhibit hyperoxaluria, a detailed clinical history is likely to reveal whether the patient has secondary hyperoxaluria. In our patient with tetraplegia who was demonstrated as having hyperoxaluria, the history of massive bowel resection directed us to consider the diagnosis of short bowel syndrome and secondary hyperoxaluria. When a clinical history is not available, measurement of plasma oxalate and of urinary glycolate would help to distinguish between primary and secondary hyperoxalurias. If secondary hyperoxaluria is suspected, the stool should be examined for *O. formigenes*, an intestinal oxalate-degrading bacterium, as lack or absence may lead to increased intestinal oxalate absorption. The next diagnostic step is to study the intestinal oxalate absorption using $[^{13}C_2]$oxalate. Therapeutic measures to prevent recurrence of calcium oxalate nephrolithiasis include: (1) a diet low in oxalate, but normal or high in calcium; (2) a high fluid intake (>1.5 $l/m^2$/day); (3) oral citrate supplementation to increase the urinary oxalate solubility; (4) specific treatment in patients with malabsorption syndromes, depending on the underlying pathology; and (5) intestinal recolonization of *O. formigenes* or the treatment with other oxalate-degrading bacteria[20].

In future, oral administration of *O. formigenes* or lactic acid bacteria may prove to be a promising new therapeutic tool in patients with primary and secondary hyperoxaluria. Lactic acid bacteria are currently classified as nonpathogenic bacteria, which are permitted in food by the U.S. Food and Drug Administration. However, caution may be required when probiotic preparations are used in immunocompromised patients[21].

## ACKNOWLEDGMENTS

The authors wish to express profound thanks and gratitude to TSW Urology Editor, Dr Ziya Kirkali for the valuable help and guidance. The leading author is grateful to Dr. Harmeet Sidhu and Dr. Albert Fosmoe, Oxalate Division, Ixion Biotechnology, Inc., Alachua, FL, for their valuable help.
